# Vocational Training for General Practice

**Published:** 1968-10

**Authors:** Michael Lennard


### VOCATIONAL TRAINING FOR GENERAL PRACTICE

BY

MICHAEL LENNARD

*" Education is not that one knows more, but that one behaves differently."*
—Ruskin.

*" If the General Practitioner of the future were a man who had done five years post-graduate training in hospital, there is no doubt that the whole status of General Practice would improve, and the public would have access to a higher quality of medical care."*
—Sir Robert Platt.

### PAST AND PRESENT

At no time since the middle of the last century has there been such a ferment on the subject of medical education as there is at present. Then the discussion was about standards of qualification to call oneself a doctor and to practise; 1858 and the formation of the General Medical Council heralded a recognizable and acceptable minimum level of training; in 1886 a triple qualification became compulsory, and from then on the holder of such a qualification was adjudged " fit to practise ". Not until the Medical Act of 1950 was this basic concept altered, and then the law decreed " a satisfactory year of general vocational training " as necessary for all medical graduates.

During the last hundred years medical science has made enormous progress, and has made necessary the establishment of a great number of specialties within medicine. The three main specialties, and many of the more recent ones have so organised and set their standards that the young intending specialist has from the outset of his career a clear idea of what he must do, and what standard he is expected to achieve; he has also a fair knowledge of what, as he climbs his career ladder, his rewards will be. Only for general practitioners, numerically the largest group in medicine, is there no accepted career structure for the early training years. The greater proportion of intending G.P.'s do try to train themselves, but they do this knowing that their ultimate reward will be unlikely to be affected, however long and careful their training.

The moment that it was accepted that undergraduate medical education does not produce a doctor ready for practice, but only a undifferentiated but totipotential ' fœtal ' physician, then the whole former basis of undergraduate education went into the melting pot. The University medical schools, wishful to free the undergraduate curriculum of some of its load, were yet faced with a dilemma. As John Ellis (1963) put it, in discussing some of the problems of education, one difficulty

> " has been the failure . . . to make the pre-registration year a proper general vocational training. Another has been the failure . . . to demand and provide a post-graduate training for general practitioners. Until this is done, 50% of our medical graduates will continue to go into practice without adequate preparation—what is worse the undergraduate education of all our doctors will continue to suffer because as no one knows which 50% of the students will get a seven year postgraduate training and which will not, the hopeless attempt to give comprehensive coverage to all will inevitably continue."

It is in this context that one must consider the present state of vocational training for general practice, and its future needs, bearing in mind that this training should be involving nearly a half of the medical graduates qualifying each year.

To consider in true perspective the proper vocational training of the G.P. one must have a clear notion of the content of general practice and of the special function of the general practitioner. The College of General Practitioners has produced a definite statement of their views on this matter (Lancet 1962) which goes some way to satisfying everybody as far as this is possible. McWhinney (1966) in a paper on ' General Practice as an academic discipline ' gives, as the criteria to be fulfilled by such a discipline,

1. A unique field of action,
2. A defined body of knowledge,
3. An active area of research,
4. A training which is intellectually rigorous.

Under the first heading, McWhinney says, " The G.P.'s sphere of pre-eminence can be defined in one sentence: ' The detection of the earliest departures from normal '." The body of knowledge he defines under five broad headings :—

1. The evaluation of symptoms, signs, and diagnostic tests.
2. The prevalence, incidence, distribution, and natural history of disease.
3. The physical and mental development of human beings from intra-uterine to adult life.
4. Human behaviour and psychodynamics.
5. Social influence in the presentation of disease.

The last criterion—an intellectually rigorous training—is, he says, ' the one which has been most neglected in Great Britain '.

The prevention and early diagnosis of disease, then, offer a challenge which the G.P. is, before all his medical colleagues, best placed to meet. But training for this task in the 1970's must be adequate for longer than a decade; the men training now are looking forward to active careers of about forty years, therefore it is necessary to suggest, on the basis of present trends, some changes which may affect practice at least over the next one or two decades.

(1) It is clear that there will be an increasing tendency towards the ' team-concept ' in general practice, involving medical, paramedical and non-medical workers. This envisages an increasing co-operation between the general medical services and those of the public health department.

(2) Interest in preventive medicine in its widest sense is growing and this will continue. Health education, specific prevention, and pre-symptomatic diagnosis will assume a more important place—and occupy a greater amount of time, and here the team-concept will be important.

(3) Our therapeutic range will continue rapidly to increase. This will enlarge the scope of the G.P. in the field of medical care in the home (which incidentally may prove an economic boon to the N.H.S.). This trend is already shown in the acute infections and in the care of the mentally sick.

(4) The growing importance of the behavioural sciences to the doctor is of importance.

(5) It may prove that the current demand for the G.P. to have hospital beds will increase, and certainly he must have full and open access to all diagnostic facilities.

In brief, the objective of vocational training for general practice is to produce a young graduate who, having made a *positive* choice of a career in family practice, has followed a planned course of training to fit him to start that career as an independent practitioner, and who is aware that education is a lifelong process. This course of training should have a wide clinical range with particular emphasis on the earliest diagnosis of disease. Clearly this can initially be taught most easily in hospitals where suitable clinical material can be concentrated. But it requires teachers aware of the needs of the trainee; complex technical skills required by the specialists may not be necessary to the G.P., for while the latter needs to know the *potential* of his specialist colleague he does not need all his expertise. Vocational training starts with the pre-registration year. The two appointments in this year—presumably internal medicine and surgery—are an integral part of the training programme and it is vitally important that they achieve what this experience is meant to achieve—a sound basis of general vocational training in medicine. The paper by Hutton *et al*, on " The Pre-registration House Appointment " (1964) suggests that this object is by no means universally attained.

Further hospital training in other disciplines is necessary, but clearly the G.P. trainee cannot spend time in hospital over every discipline in the field of medicine. He must concentrate on those subjects in which training is of greatest value. The work of Peterson *et al* (1956) strongly suggests that internal medicine is the most important. Perhaps one might add pædiatrics as being a basically similar discipline. Obstetrics is clearly a vital experience, perhaps combined with gynæcology, with, it is to be hoped, a strong bias towards antenatal care and the prevention of trouble in pregnancy, labour, and the puerperium. Psychiatry is important but most psychiatric hospital jobs are hardly suitable; those in general hospital psychiatric departments are better, as they carry a larger out-patient load of less severe disability—but such jobs are few in number. Other subjects lend themselves to shorter appointments, perhaps on a rotating basis. But no trainee can hope to cover the whole field of clinical experience by in-service training; it will be necessary for him to fill the gaps either as a clinical (out-patient) assistant, perhaps during his G.P. trainee year or even after, or by special courses. In all this, there is implicit the need for the supervising specialist to be aware that the particular trainee's aim is family practice, and to fulfil his teaching function in this situation he must himself have an awareness of, and a respect for, the function of the G.P., and the considerable area of general medical work that is outside normal specialist experience. He should be aided in his task by teachers drawn from local general practice.

While the trainee will not be, of course, fully trained in the clinical content of general practice after this hospital post-graduate training, he should be competent enough in straight-forward clinical examination and have enough judgement to be ' safe ' away from the facilities and staff of the hospital. He will have much to learn in the ' elucidation of undifferentiated clinical problems ' and he will have to adapt his knowledge to a new setting. In addition he must learn to think in terms of total health care—a much bigger concept than the episodic medical care of the hospital, for it implies a new dimension of responsibility to individuals, to families, and to the community in which his work lies. In addition to clinical work, the young graduate must learn about the social complications and implications of disease, about industry and the problems of work and rehabilitation, about organisation, about ethics and law, and (hopefully) about research, or at least the potential of research in general practice.

Ideally initial experience of this sort should be gained, like hospital experience, under the *active* supervision of well qualified and experienced teachers in the subjects in question—in short from established G.P.'s, with special experience in this sort of teaching. The official G.P. Traineeship scheme as instituted in this country is, at least theoretically, the ideal answer to the problem of this phase of training. In fact this ideal is not always—or even often—achieved; much could be done to make the excellent idea of the trainee year live up to its promise. Just as the hospital training period tends to lack a planned programme of education, so does the trainee year; but given this, and proper academic supervision, it could become a really worthwhile scheme. Close co-operation with the medical school and the hospital would be important, for the traineeship year should contain opportunities for regular sessional hospital work in those disciplines not covered in the hospital training period.

Implicit in the reforms and innovations mentioned is the need for an experienced, competent, and enthusiastic body of G.P. teachers. Reforms that fail to develop the latent potential among established practitioners, or that forget to train the teachers of tomorrow, can do little more than disturb the fringe of the problem.

The whole problem of G.P. training is not only real, it is also urgent. It is not beyond the bounds of very real possibility that the profession as a whole may suddenly find itself faced with a considerable demand for planned education. This would have to be met at first by existing facilities, establishments, and institutions, that is, the present medical schools, hospitals, and practices that are already deeply committed to their function of service. Yet they must accept, and learn to shoulder, the demands that will be made on them.

### FUTURE

The aim, then, for the future is the firm establishment of an accepted and planned career structure for post-graduate vocational training for general practice; accepted, that is, by the profession in general as being as necessary as post-graduate training for any other branch of medicine, and in particular by the young graduate wishing to become a G.P.; planned in the sense that there should be a guaranteed and reasonably uniform standard and content to the training. Reasonable basic proposals along acceptable lines have been made by the Royal College of General Practitioners, by the G.M.S.C., by the B.M.A., and the whole general idea has been blessed in the recent reports of the G.M.C. and of the Royal Commission on Medical Education.

One can, however, say in advance that no scheme will succeed unless certain conditions are fulfilled.

(1) There must be a sufficient number of hospital posts specifically for G.P. training. Individual posts, or groups of posts at particular hospitals, should be organised to give suitable experience; and there is scope here for hospital management boards to consider rotating or combined appointments. Further, training posts must fulfil requirements and accept inspection. Only in this way can trainees—and any interested authority—be sure that the necessary training is being given.

(2) There must be an adequate supply of teachers in general practice both to supplement the hospital training, and to provide the training in practice. This will of necessity involve large numbers of G.P.'s and will entail a programme—and perhaps an urgent one—for training the trainers.

(3) There must be, if not actual financial incentives, at least a lack of disincentive. The trainee must know that his following the accepted career training will not penalise him financially, he must be on equal terms with those training for other branches, he must be satisfied that the training he is taking will 'pay a dividend' ultimately, *vis-a-vis* those who do *not* take it; and if trainers are to give the right and necessary attention and time to their training commitments, they, too, must be adequately rewarded.

These things imply the need for some sort of overall authority governing the G.P. training programme in the country. This authority would ideally have some sort of central body, but with regional 'councils' possessing a reasonable degree of autonomy. The regions could conveniently coincide with the Regional Hospital Boards and Postgraduate Medical Advisory Councils. It has been suggested that each such region and council would need a 'General Practice Postgraduate Adviser' who, part-time or full-time, would act as its working agent. The functions of this council and its adviser could be to help students and pre-registration graduates in their choice of careers; to advise and direct those making a positive choice of general practice on the training they should take and on *approved* facilities in the region; to advise and assist hospitals anxious to fulfil the necessary criteria for their resident posts; and to hold a watching brief over practice traineeships. It should help in the planning and organisation of the necessary *formal* courses in the region, and promote the formation of the local *informal* study groups both for the hospital trainee, and for his fellows in trainee practice. It should set up in the region a network of 'teaching practices' (which might or might not be traineeship practices) whose principals accept the need for their involvement in training at all the various levels. Finally it should promote the fullest possible liaison between all concerned in the education and training of young G.P.'s. This might entail organisation similar to the present system of area clinical tutors, which would mean 'area G.P. tutors,' perhaps from one of the 'network practices'.

The above outline is in accordance with all the various reports on the subject including the Todd report. The transformation is going to require effort, good-will, and money. The money will I believe be forthcoming from the only possible source, the Treasury, if the profession shows enough co-operation and goodwill in all its branches to make it clear that the *whole* profession is behind the demand for proper facilities for G.P. vocational training. But this co-operation must be active and energetic, for goodwill by itself will not be enough.

Given these things, the money will come; the only question then will be through what channel it will be administered. Two possibilities and only two really exist; either the money will come through the Regional Hospital Boards, or through the Universities. There may be advantages and disadvantages both ways from an administrative point of view, but there is in my opinion little doubt that from an academic point of view the University Medical School should be the supervising body. Is Bristol prepared to take this on, for its own clinical area at least, and, ideally, for the whole South West region? Let there be no mistake; involvement will mean going the whole way, up to and including a department of general practice within the medical school, which could properly set the necessary standards. It would, and this can be firmly promised, be assured of the full co-operation of all the general practice organisations in the region; it would also need equal co-operation from all specialists, both on a hospital and an individual basis.

The effects of these changes on the standards of medical care in this area could be of great benefit to the whole region; the possibilities of benefit to the undergraduates teaching programme of an expanding medical school should not be forgotten. But many experienced doctors, not by nature pessimistic or cynical, doubt the Universities' ability to descend from their traditional ivory towers and to concern themselves seriously with medicine outside the hospitals. Are they right?

### REFERENCES

College of General Practitioners (1965) "Special Vocational Training for G.P."

Ellis, J. (1963) *Brit. Med. J.*, **ii**, 585.

Hutton *et al.* (1964) *Lancet* **i**, 38.

Lancet (1962) **ii**, 1108.

McWhinney, I. R. (1966) *Lancet* **i**, 419.

Peterson *et al.* (1956) *J. Med. Ed.* (*Suppl.*) 37,72 (2).

Platt, Sir R. (1963) '*Doctor and Patient*'—Nuffield Provincial Hospital Trust.

